# Myo-inositol elevation as an *in vivo* marker of reactive gliosis in pediatric Friedreich ataxia: evidence from HERMES-edited MR spectroscopy

**DOI:** 10.1016/j.nicl.2026.103983

**Published:** 2026-03-03

**Authors:** William Gaetz, Muhammad G. Saleh, Charlotte Birnbaum, Luke Bloy, Timothy P.L. Roberts, David R. Lynch

**Affiliations:** a Department of Radiology, Children’s Hospital of Philadelphia and University of Pennsylvania, Philadelphia, PA, USA; b Department of Neurology, Children’s Hospital of Philadelphia and University of Pennsylvania, Philadelphia, PA, USA

**Keywords:** Friedreich ataxia (FRDA), Magnetic resonance spectroscopy (MRS), HERMES editing, Glutathione (GSH), γ-Aminobutyric acid (GABA), Myo-inositol (mI)

## Abstract

**Background:**

Friedreich ataxia (FRDA) is a rare neurodegenerative disorder caused by frataxin deficiency and is characterized by mitochondrial dysfunction, oxidative stress, and progressive motor dysfunction. Most *in vivo* MRS work in FRDA has focused on the cerebellum, brainstem/pons, and spinal cord, consistently reporting abnormalities in the neuronal marker N-acetylaspartate (NAA) and the glial metabolite myo-inositol (mI). To our knowledge, the NAA/mI ratio in the primary motor cortex has not been reported in FRDA, particularly in pediatric cohorts. Additionally, *in vivo* edited MRS measurements of the inhibitory neurotransmitter γ-aminobutyric acid (GABA+ (GABA + macromolecular contributions)) in FRDA have not yet been reported and GSH has been examined only rarely in FRDA and, to our knowledge, has not been studied in the motor cortex in either adult or pediatric cohorts.

**Objective:**

To assess GSH, GABA+, NAA, and mI across cerebellum and motor cortices in pediatric FRDA using HERMES-edited MRS.

**Methods:**

We acquired HERMES MRS data from 16 children with FRDA and 15 age-matched controls. Tissue-corrected metabolite estimates were obtained using LCModel and voxel-based tissue segmentation. Linear mixed models (LMMs) were used to evaluate group and region effects, with subject as a random effect.

**Results:**

LMMs revealed no significant group differences in tissue-corrected GSH or GABA + . In contrast, the tNAA/mI ratio was significantly reduced in FRDA (p < 0.001), driven by elevated mI (p < 0.001), while tNAA did not differ between groups (p = 0.150). ROI-specific analyses showed higher mI in FRDA in both motor cortices after Bonferroni correction, with a non-significant trend in cerebellum (pcorr = 0.054).

**Conclusions:**

These findings support a model of early reactive gliosis in pediatric FRDA, indexed by elevated mI and occurring without statistically significant neuronal loss, (acknowledging that significant reductions in tNAA may require larger samples to resolve), and extend prior cerebellar-focused work to the primary motor cortex. While GSH and GABA + did not differ between groups, the observed mI elevations highlight myo-inositol as a practical *in vivo* biomarker of astrocytic activation and a candidate marker for disease progression in FRDA. Longitudinal studies are needed to confirm its sensitivity to clinical trajectory and therapeutic response.

## Highlights

• First *in vivo* glutathione (GSH) and GABA measurement in pediatric Friedreich ataxia using HERMES-edited MRS.
• No significant group-level differences in GSH or GABA+, despite strong rationale for oxidative and inhibitory dysfunction in FRDA.
• Significant reduction in the tNAA/mI ratio in FRDA, driven by elevated myo-inositol rather than reduced tNAA (tNAA not significant, p = 0.150).
• Myo-inositol elevations are significant in both motor cortices (Bonferroni-corrected), with a trend in cerebellum (pcorr = 0.054).

## 1 Introduction

Friedreich ataxia (FRDA) is a rare, autosomal recessive neurodegenerative disorder caused by GAA trinucleotide repeat expansions in the *FXN* gene, resulting in reduced expression of the mitochondrial protein frataxin (Pandolfo, 2009, Cook and Giunti, 2017, Campuzano et al., 1996). Frataxin deficiency impairs mitochondrial function, disrupts iron-sulfur cluster biogenesis, and leads to oxidative stress and progressive neuronal degeneration (Cook and Giunti, 2017, Martelli et al., 2007). Clinically, FRDA is characterized by early-onset gait and limb ataxia, dysarthria, loss of proprioception, and progressive cardiomyopathy, features that often emerge in childhood or adolescence and worsen over time (Pandolfo, 2009, Tsou et al., 2011, Durr et al., 1996). Despite advances in genetic characterization, there remains a critical need for non-invasive, *in vivo* biomarkers capable of tracking disease progression and evaluating therapeutic response (Georgiou-Karistianis et al., 2025).

**Fig. 1:**
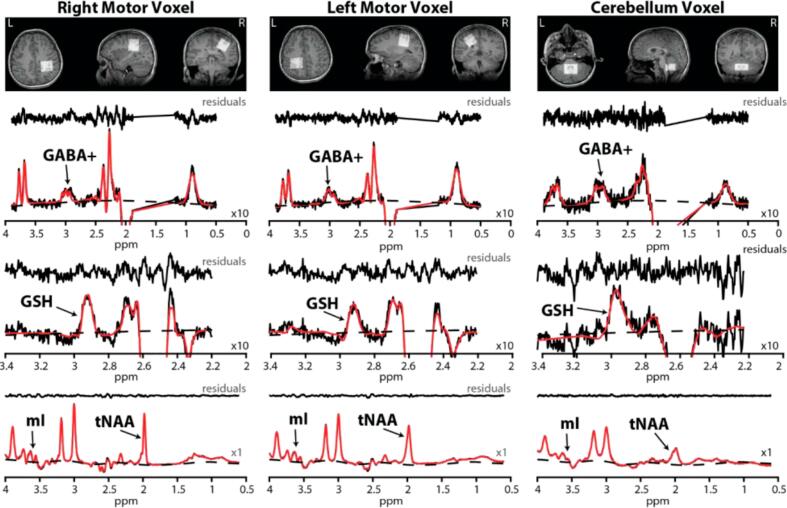
Voxel placement and representative HERMES-edited spectra from right and left motor cortices and cerebellum. T1-weighted MRI slices (axial, sagittal, coronal) show voxel placement in right M1, left M1 (30 × 30 × 30 mm^3^), and midline cerebellum (30 × 30 × 20 mm^3^). Corresponding spectra (black) from each region include the GABA-edited difference spectrum (arrow showing the 3.0 ppm GABA + peak) and GSH-edited difference spectrum (arrow showing 2.95 ppm GSH peak), and sum spectrum (arrows showing mI and total NAA, tNAA) modeled (red) with baselines (broken lines) in LCModel. *Note:* A × 10 scaling factor was applied to GABA + and GSH for visualization purposes; mI and tNAA were plotted at native scale (×1). (For interpretation of the references to colour in this figure legend, the reader is referred to the web version of this article.)

Magnetic resonance spectroscopy (MRS) has been used in FRDA to detect neurochemical alterations in the central nervous system, including reduced N-acetylaspartate (NAA; neuronal marker) and elevated myo-inositol (mI; glial marker) in the cervical spinal cord (Joers et al., 2022) and in the cerebellar vermis/hemispheres and pons (Iltis et al., 2010). Prior work in the cerebellar hemispheres and pons using short-TE MRS at 4 T has reported alterations in NAA and mI, summarized as a reduced NAA/mI ratio, and has also suggested pontine GSH abnormalities, including an elevated GSH/tNAA ratio in FRDA (Iltis et al., 2010). To our knowledge, the NAA/mI ratio in the *primary motor cortex* has not been reported in FRDA, particularly in pediatric cohorts. Beyond these conventional markers (and their ratios), there is strong rationale to expand the neurochemical profile to include glutathione (GSH) and γ-aminobutyric acid (GABA): GSH is the brain’s primary antioxidant and an *in vivo* indicator of oxidative stress, central to FRDA pathogenesis, yet brain GSH by MRS has been examined only rarely in FRDA and, to our knowledge, has not been studied in the motor cortex in either adult or pediatric cohorts (Piemonte et al., 2001). GABA, the main inhibitory neurotransmitter critical for motor control, may reveal altered inhibitory tone with edited MRS (Petroff, 2002). Together with established measures like NAA/mI, GSH and GABA could provide a more comprehensive view of FRDA pathophysiology and improve sensitivity for tracking progression and treatment response (Fig. 1).

Hadamard Encoding and Reconstruction of MEGA-Edited Spectroscopy (HERMES) is an advanced “edited” MRS technique developed to enable simultaneous detection of multiple low-concentration metabolites, such as GABA and GSH, in a single scan. In the method described by Saleh et al. (NeuroImage, 2016), HERMES applies a Hadamard encoding scheme to interleave multiple MEGA editing pulses across four sub-experiments. The Hadamard combination of sub-spectra isolates the signals of interest, GABA and GSH (Saleh et al., 2016). Although HERMES is designed primarily for edited detection of low-concentration metabolites like GABA and GSH, the acquisition also yields high-quality sum spectra that can be used to quantify conventional metabolites such as NAA and mI. Previous studies have demonstrated that reliable quantification of abundant metabolites such as NAA and mI is feasible, despite their tissue relaxation time constant (T2) values, from longer-TE summed spectra, particularly when LCModel is used in combination with simulated basis sets and optimized spectral modeling (Saleh et al., 2016, Kreis and Ross, 1993, Oeltzschner et al., 2020, Saleh et al., 2019). In this study, we leveraged the sum spectra from HERMES to derive NAA and mI estimates, ensuring internal consistency across all acquisitions and subjects while enabling simultaneous examination of both canonical and edited metabolites within the same voxel.

Assessments of GSH and GABA in the cerebellum and motor areas of FRDA patients using *in vivo* MRS are notably absent from the current literature. While GSH is a key antioxidant involved in counteracting oxidative stress, a core feature of FRDA pathology, and GABA is central to inhibitory neurotransmission (Petroff, 2002), *in vivo* measurements of these metabolites in FRDA have been limited, with prior GSH findings restricted primarily to brainstem regions and adult cohorts (Iltis et al., 2010). In this study, we addressed this gap by using edited MRS to quantify GSH and GABA levels in the cerebellum, where pathology is well established (Iltis et al., 2010), and in the primary motor cortex, which is increasingly recognized as a site of disease involvement (Georgiou-Karistianis et al., 2025). We hypothesized that *in vivo* levels of both GSH and GABA would be reduced in proportion to clinical severity.

## 2 Methods

### 2.1 Ethics

This study was approved by the Institutional Review Board of the Children’s Hospital of Philadelphia (IRB# 23–021822). All procedures conformed to the Declaration of Helsinki and HIPAA regulations. As participants were minors, written parental/guardian consent was obtained for all subjects and child assent was obtained when age-appropriate (≥7 years, per IRB policy). MRI/MRS procedures were noninvasive, and all data were de-identified prior to analysis.

### 2.2 Inclusion criteria

**Fig. 2:**
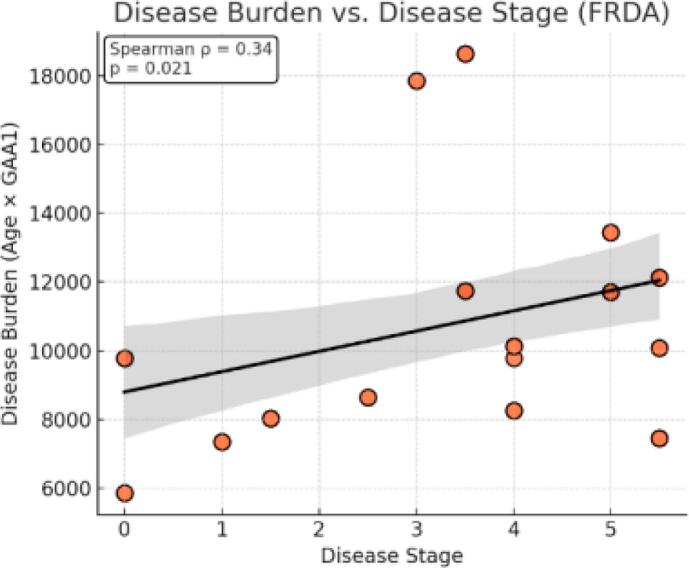
Disease Burden vs. Disease Stage Our FRDA sample included individuals across a range of clinical stages. We observed a significant positive correlation between Disease Burden (defined as the product of age and GAA1 repeat length) and Disease Stage (Spearman ρ = 0.34, p = 0.021). These findings are consistent with the progressive nature of FRDA, in which cumulative genetic and developmental load increases with advancing clinical stage.

FRDA patients between the ages of 8 and 15 years were of specific interest for recruitment, as they were unexposed to omaveloxolone (FDA approved for individuals with FRDA > 16 years of age) (Fig. 2).

### 2.3 Data acquisition

**Fig. 3:**
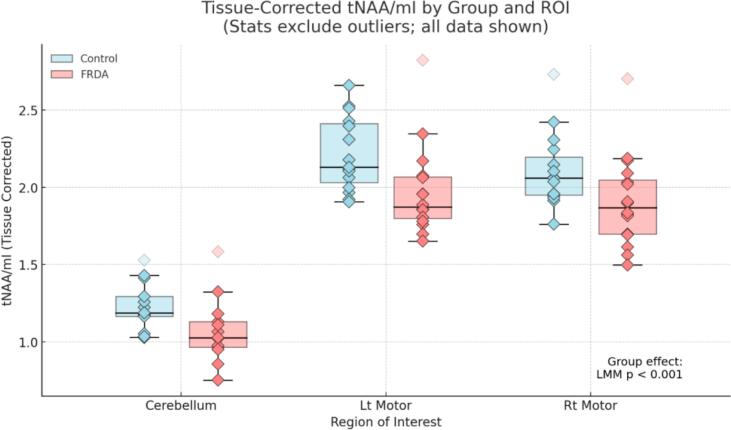
Group differences in tissue-corrected tNAA/mI ratios across brain regions.

MR imaging (MRI) and MRS experiments were conducted on a 3 T Siemens Prisma scanner using a 32-channel head coil. MRS was performed in three regions: cerebellum (where the voxel was placed to straddle the midline and thus capture both dentate nuclei), and the left motor cortex and right motor cortex with voxels centered on the precentral motor ‘hand knob’ anatomical landmarks. Before every MRS acquisition, a high-resolution 1 mm^3^ whole-brain T_1_ structural image was acquired to aid with the voxel placement. The MRS experiments involved using the Hadamard Encoding and Reconstruction of Mega-Edited Spectroscopy (HERMES) sequence (see Saleh et al., 2019). The multi-vendor standardized HERMES sequence was designed with the slice-selective and frequency-selective RF pulses for PRESS localization and editing. The duration of the slice-selective excitation (asymmetric sinc-Gaussian) and refocusing pulses were 7.2 ms (BW = 2247 Hz) and 7.0 ms (1342 Hz), respectively, both with peak B1 13.5 µT. The HERMES editing scheme has four sub-experiments (A, B, C, and D) for the simultaneous detection of GABA at 3.0 ppm and GSH at 2.95 ppm. The following HERMES editing scheme comprised four sub-experiments: A) a dual-lobe editing pulse (ON_GABA_ at 1.9 ppm; ON_GSH_ at 4.56 ppm); B) a single-lobe editing pulse (ON_GABA_); C) a single-lobe editing pulse (ON_GSH_); and D) a single-lobe editing pulse at 7.5 ppm (OFF_GABA/GSH_) (Saleh et al., 2016, Saleh et al., 2019). Hadamard combinations A + B–C–D yielded GABA-edited spectra, A–B + C–D yielded GSH-edited spectra, and A + B + C + D yielded the sum spectra. GABA-edited spectrum detects the GABA + macromolecule signal at 3 ppm (GABA + from here on). HERMES data in the right and left motor cortices were acquired using the following parameters: 30 × 30 × 30 mm^3^ voxel size; TE/TR = 80/2000 ms; 20-ms editing pulse duration; 2048 data points; 2 kHz spectral width; 320 transients; and WET water suppression. For the cerebellar region, the same parameters were used except for 30 × 30 × 20 mm^3^ voxel size. Outer-volume suppression pulses were applied to saturate spins outside the volume of interest and reduce signals from outside the voxels. Finally, unsuppressed (water) acquisitions were performed using the above parameters, except that 16 transients were collected from every region. The total scan time per region was about 12 min (Fig. 3).

### 2.4 Data processing

Data were processed in Osprey (Oeltzschner et al., 2020). The 3.02-ppm total creatine (tCr = creatine + phosphocreatine) signal was used to estimate magnetic field (B_0_) drift in the *in vivo* data before frequency-and-phase correction (FPC). Within every MRS dataset, any transients with a B_0_ drift exceeding 5 Hz were removed. Finally, FPC was performed on the remaining transients of every MRS dataset. The fully processed sub-spectra were combined to generate the GABA-edited, GSH-edited, and sum spectra. The residual water signal was removed using an HSVD filter (Barkhuijsen et al., 1987).

### 2.5 Data modeling

The HERMES data were modeled using LCModel (Version 6.3-1R) (https://s-provencher.com/lcmodel.shtml). 2D localized density-matrix simulations were performed using the HERMES parameters in MRSCloud (Hui et al., 2022) to generate LCModel basis sets as described in the previous publication (Saleh et al., 2023). The following metabolites were included in the generation of the basis sets: alanine, ascorbate, aspartate, creatine, phosphocreatine, GABA, glycerophosphocholine (GPC), GSH, glutamate (Glu), glutamine (Gln), glycine, myo-inositol (mI), lactate, N-acetyl aspartate (NAA), N-acetylaspartylglutamate (NAAG), phosphocholine (PCh), phosphocreatine (PCr), scyllo-inositol, and taurine. The water-scaled GABA + estimates were obtained from the GABA-edited spectra modeled between 0.5 and 4 ppm, with the additional LCModel control parameters: “*sptype = mega-press-3*”; “*nobase = F”*; baseline knot spacing = 0.55; and “*dows = T*”. The water-scaled GSH estimates were obtained from the GSH-edited spectra modeled between 2.2 ppm and 3.4 ppm with a knot spacing of 0.55. In order to enable water scaling in LCModel, we require a singlet in the simulated GSH-edited basis set. However, the GSH-edited spectrum does not have a singlet. Therefore, a singlet at 3.3 ppm (devoid of any *in vivo* signal) was simulated using the HERMES parameters, but without the editing pulses (sub-experiment D) and was added to the basis functions to enable water scaling of the GSH-edited spectra. The water-scaled estimates of total NAA-acetyl compounds (NAA + NAAG, tNAA), total creatine (creatine + phosphocreatine), total choline (tCho), and Glu came from the sum spectra modeled between 0.6 and 4 ppm using the default LCModel parameters as described in the previous publication (Saleh et al., 2023).

### 2.6 Quality control (QC)

Every subject’s modeled edited and sum spectra were visually assessed, and those with spurious signals (from outside the voxels), baseline distortions or the absence or poorly defined signal at expected resonance frequencies (e.g., GABA + at 3.0 ppm) were removed from further analysis. The T1-weighted images were segmented to calculate gray matter (GM), white matter (WM), and CSF voxel tissue fractions. Water-scaled concentrations were corrected for partial volume and relaxation effects in each voxel using the method described by Gasparovic and colleagues (equation in *OspreyQuantify*) (Gasparovic et al., 2006). The values for T1/T2 of GABA were 1310/88.5 ms, GSH were 1265/116.75 ms, tNAA were 1337.5/225.9 ms, and mI were 1120/198.7 ms. The following tissue-specific relaxation times for water were used: T1/T2 of GM were 1331/110 ms, WM were 832/79.2 ms, and CSF were 3817/503 ms.

Data quality indices (LCModel SNR and spectral linewidth, FWHM) are summarized in Table 1. Cerebellar spectra showed lower SNR and broader linewidths relative to motor cortex; however, there were no significant group differences in SNR or FWHM within any ROI (all p ≥ 0.15), indicating comparable spectral quality between FRDA and control participants.

**Table 1:** Signal-to-noise ratio (SNR) and spectral linewidth (FWHM, Hz) are reported for each ROI and diagnostic group. Cerebellar spectra show lower SNR and broader linewidths than motor cortex, but values are comparable across groups within each ROI. Sample size (n) reflect the datasets passing quality control (QC).

ROI	Group	n	SNR (mean ± SD)	FWHM (Hz, mean ± SD)
Left Motor	Control	15	38.9 ± 6.19	4.73 ± 1.34
	FRDA	16	38.1 ± 3.21	4.73 ± 1.22
Right Motor	Control	15	38.1 ± 4.71	5.00 ± 1.95
	FRDA	16	34.6 ± 8.14	5.03 ± 1.97
Cerebellum	Control	14	36.1 ± 14.13	6.54 ± 2.96
	FRDA	14	34.1 ± 9.51	6.95 ± 2.26

## 3 Results

### 3.1 Participants

Our sample included 16 children with Friedreich ataxia (FRDA) and 15 age-matched typically developing controls. The mean age of the control group was 12.5 years (SD = 1.8, range = 9.9 to 16.0), while the FRDA group had a mean age of 11.9 years (SD = 1.8, range = 8.9 to 15.3). The sex distribution was similar across groups, with 7 females and 8 males in the control group and 7 females and 9 males in the FRDA group. The mean short-arm GAA repeat length in the FRDA group was 905 (range = 560–1210). The mean disease burden, defined as the product of Age and GAA1 repeat length, was 10,800 (range = 5,850–18,600).

### 3.2 MRS results

For edited spectra, the number of transients retained after drift removal did not differ significantly between groups. Following visual quality assessment, the remaining datasets from RT Motor/LT Motor/Cerebellum that generated GABA-edited spectra were 15/14/13 for Controls and 15/16/13 for FRDA; GSH-edited spectra: 15/15/13 for Controls and 15/16/12 for FRDA; and sum spectra (containing tNAA and mI) were 15/15/13 for Controls and 16/16/13 for FRDA.

A linear mixed model (LMM) was used to evaluate group and region (ROI) effects on tissue-corrected GSH, with subject as a random effect. The model revealed no significant effect of group (p = 0.93), indicating that overall GSH levels did not differ between the FRDA and control groups. In contrast, there was a highly significant main effect of ROI (p < 0.001), with GSH levels significantly lower in both motor cortices compared to the cerebellum, consistent with a regionally specific gradient in glutathione concentration. The group × ROI interaction was not formally tested, as the absence of a group effect and the strength of the ROI effect suggest primarily anatomical rather than disease-specific differences in GSH.

A linear mixed model (LMM) was used to evaluate group and region (ROI) effectson tissue-corrected GABA concentrations, with subject as a random effect. The model revealed no significant main effect of group (p = 0.953), indicating no global difference in GABA levels between FRDA and control participants. In contrast, there was a highly significant main effect of ROI (p < 0.001), with GABA + levels significantly lower in both motor cortices compared to the cerebellum, consistent with a strong regionally specific gradient. In contrast, an LMM revealed a significant main effect of group on the tissue-corrected tNAA/mI ratio (p < 0.001), with individuals with FRDA exhibiting reduced tNAA/mI levels relative to controls across brain regions. A strong main effect of ROI was also observed (p < 0.001), with tissue-corrected tNAA/mI ratios significantly higher in both motor cortices compared to the cerebellum. The omnibus Group × ROI interaction was not significant, indicating that the FRDA-related reduction in tNAA/mI did not differ across regions.

Box plots display tissue-corrected tNAA/mI ratios for control (light blue) and FRDA (coral) groups across three regions of interest (ROIs): Cerebellum, left motor cortex, and right motor cortex. Individual subject values are overlaid as diamond-shaped markers. Outliers were defined separately within each Group × ROI using the 1.5 × interquartile range (IQR) rule, with quartiles computed by linear interpolation; datapoints falling strictly below Q1–1.5 × IQR or above Q3 + 1.5 × IQR were flagged as outliers. These values are displayed as faded markers but were excluded from statistical testing. A linear mixed model (LMM), conducted after excluding outliers, revealed a significant main effect of group (p < 0.001), indicating a global reduction in tissue-corrected tNAA/mI among individuals with FRDA compared to controls. The group effect remained statistically significant when outliers were included (p = 0.009), confirming that the observed group difference was not driven by extreme values.

To determine whether the group-level reduction in the tNAA/mI ratio reflected neuronal loss (reduced tNAA), gliosis (elevated mI), or both, we conducted follow-up LMMs for each metabolite separately (Fig. 4A & Fig. 4B). These analyses revealed no significant group difference in tissue-corrected tNAA (p = 0.150), but a significant elevation in mI in the FRDA group (p < 0.001), indicating that the group effect on the tNAA/mI ratio was primarily driven by increased myo-inositol.

**Fig. 4A:**
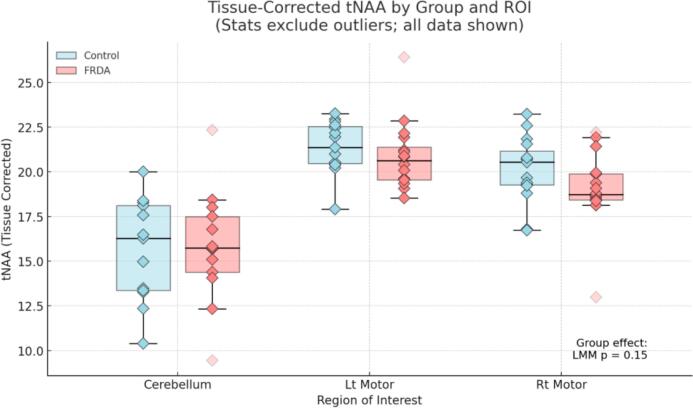
Decomposition of the tNAA/mI ratio: Tissue-corrected tNAA by group and brain region. Box plots show tissue-corrected tNAA concentrations by group (light blue = Control; coral = FRDA) for each ROI. Individual subject values are displayed as diamond markers; outliers were defined separately within each Group × ROI using the 1.5 × IQR rule and are shown as faded points but were excluded from statistical analyses. A group-level LMM revealed no significant difference in tissue-corrected tNAA between FRDA and control groups (p = 0.150). ROI-specific Welch’s t-tests likewise showed no significant differences in any region. (For interpretation of the references to colour in this figure legend, the reader is referred to the web version of this article.)

**Fig. 4B:**
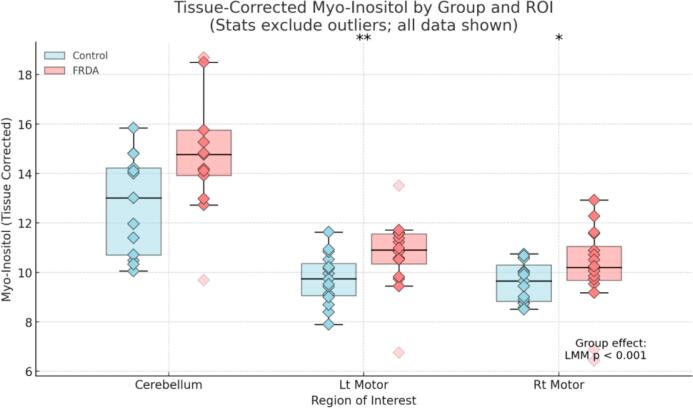
Decomposition of the tNAA/mI ratio: Tissue-corrected mI by group and brain region. Box plots show tissue-corrected mI concentrations by group (light blue = Control; coral = FRDA) for each ROI. Individual subject values are overlaid as diamond markers; outliers were defined separately within each Group × ROI using the 1.5 × IQR rule (linear quartiles), (with values exactly at the IQR fence retained) and are shown faded but excluded from statistical tests. A group-level LMM revealed a significant elevation of tissue-corrected mI in FRDA compared to controls (p < 0.001). Post hoc Welch’s t-tests with Bonferroni correction (3 ROIs) showed higher mI in FRDA in the left motor cortex (pcorr = 0.009) and right motor cortex (pcorr = 0.021); the cerebellum showed a non-significant trend (pcorr = 0.054). Asterisks indicate Bonferroni-corrected significance: p < 0.05 (*), p < 0.01 (**).

### 3.3 tNAA (motor cortex and cerebellum)

Although group differences in tNAA did not reach statistical significance in the primary linear mixed-effects model, inspection of the regional data suggested that tNAA values were numerically lower in FRDA, particularly in the motor cortices. ROI-specific Welch t-tests showed a trend toward reduced tNAA in the left motor cortex (p≈0.08; Cohen’s d ≈ 0.67), with a smaller difference in the right motor cortex (p≈0.27). In contrast, cerebellar tNAA values were nearly identical between groups. Taken together, these findings suggest that reductions in cortical tNAA may be present in FRDA, although the current study was likely underpowered to detect these effects with statistical confidence.

ROI-specific Welch’s independent-samples t-tests (Bonferroni-corrected across 3 ROIs) showed higher mI in FRDA in the left motor cortex (pcorr = 0.009) and right motor cortex (pcorr = 0.021); the cerebellum showed a non-significant trend (pcorr = 0.054), establishing that elevated mI is a regionally consistent contributor to the reduced tNAA/mI ratio in FRDA (Fig. 4A, Fig. 4B).

To assess whether increased mI was related to clinical severity, we fit linear mixed models with disease stage as a fixed effect, ROI as a covariate, and subject as a random intercept (outliers excluded by the IQR rule within ROI). Across ROIs (controlling for ROI), stage showed a positive but non-significant association with tissue-corrected mI (β = 0.19, p = 0.093). In ROI-specific LMMs, no association survived Bonferroni correction for three comparisons (left motor pcorr > 0.2; right motor pcorr > 0.2; cerebellum pcorr = 0.126). Stage × ROI interactions were not significant (p = 0.29 for left motor; p = 0.39 for right motor).

## 4 Discussion

This study provides the first *in vivo* assessment of glutathione (GSH) and γ-aminobutyric acid (GABA + ) in pediatric FRDA using multi-metabolite edited MRS. Across cerebellum and bilateral motor cortex, we identified a robust reduction in the tNAA/mI ratio driven by elevated myo-inositol. In contrast, tissue-corrected GSH and GABA + did not differ between groups. The null GSH result should be interpreted cautiously: conventional edited MRS primarily quantifies reduced GSH, whereas disease-related oxidative stress may shift the redox balance toward oxidized GSH (GSSG), which is not detected. Region-specific preservation or developmental compensation in children may also contribute, and redox-sensitive methods (e.g., GSH:GSSG) and longitudinal designs are warranted. Notably, Iltis et al. (2010) reported an elevated GSH/tNAA ratio in the pons of adults with FRDA (Iltis et al., 2010). The absence of a corresponding GSH elevation in the present study may therefore reflect differences in region of interest, participant age, or both. Collectively, these findings highlight mI as a practical *in vivo* marker of astrocytic activation and gliosis in pediatric FRDA.

The reduction in the tNAA/mI ratio, largely attributable to increased mI rather than significantly reduced tNAA, supports a model of reactive gliosis in response to progressive neurodegeneration. Myo-inositol is highly concentrated in astrocytes, and its elevation likely reflects astrocytic activation in the setting of neuronal stress, particularly within the motor cortex and cerebellum. These findings align with histopathological evidence of astrocytosis in FRDA and with the broader interpretation of mI as a marker of glial response to neurodegeneration (Koeppen et al., 2007, MacMillan et al., 2019). Importantly, the elevation of mI in motor regions occurred in the absence of significantly reduced tNAA, suggesting a temporal dissociation between early astrocytic responses and later neuronal loss. Nonetheless, numerical reductions in tNAA were observed in the motor cortices, raising the possibility that subtle cortical tNAA abnormalities may emerge with greater statistical power or larger samples. Reactive gliosis, indexed by increased mI, is widely recognized as an early response to cellular dysfunction, whereas neuronal loss (reflected by reduced tNAA) may emerge only after prolonged progression. This temporal pattern is consistent with findings from other neurodegenerative conditions such as multiple sclerosis (MS) (Srinivasan et al., 2005), amyotrophic lateral sclerosis (ALS) (Pioro, 2000, Kalra, 2019), and Huntington’s disease (Ciarmiello et al., 2017, Sturrock et al., 2010), where elevated mI often precedes or occurs independently of reductions in NAA. Such cross-disease parallels reinforce the potential of mI as a sensitive, early biomarker of disease progression in FRDA.

We fit LMMs with disease stage as a fixed effect, ROI as a covariate, and subject as a random intercept, excluding outliers by the 1.5 × IQR rule within ROI. Across ROIs (controlling for ROI), stage showed a positive but non-significant association with mI (β = 0.19, p = 0.093). In ROI-specific LMMs, no association survived Bonferroni correction for three comparisons; the strongest nominal effect was in the cerebellum (β = 0.35, p = 0.042; pcorr = 0.126). Stage × ROI interactions were not significant (p = 0.29 for left motor; p = 0.39 for right motor). Overall, these analyses suggest insufficient power to detect region-specific stage effects at corrected thresholds. To ensure consistency across metabolites and account for regional differences in voxel composition, we applied tissue correction to all quantified metabolites, including edited GABA + and GSH. This correction is particularly important in pediatric cohorts with potential structural variability, and our approach follows best practice recommendations for edited MRS (Gasparovic et al., 2006, Harris et al., 2015).

Taken together, these data support myo-inositol as a practical, early *in vivo* biomarker of astrocytic activation in pediatric FRDA, and a candidate marker for disease progression and treatment response. By demonstrating significant myo-inositol elevations and a reduced tNAA/mI ratio in the primary motor cortex, this study extends FRDA MRS findings beyond cerebellum and brainstem and provides the first motor-cortex, *in vivo* biomarker evidence in pediatric FRDA.

### 4.1 Limitations

While our findings expand the neurochemical profile of FRDA beyond conventional neuronal and glial markers, several limitations warrant consideration. First, the sample size was modest, limiting power to detect subtle region-by-group interactions or metabolite–clinical associations, and possible group differences in tNAA. Second, analyses were restricted to three a priori regions of interest (cerebellum and bilateral motor cortices). Although chosen for their established neuropathological involvement in FRDA, the absence of a neutral control region limits assessment of regional specificity. Third, while CSF-based tissue correction reduces dilution effects, residual partial-volume variability likely remains due to differences in gray–white matter composition and local anatomical heterogeneity, which is especially pronounced in the cerebellum. Fourth, GSH detection with MRS is constrained to the reduced form, leaving potential oxidative imbalance (GSSG elevation) undetected. Complementary methods are needed for a full redox profile. Fifth, while the tNAA/mI ratio was significantly reduced, this effect appeared to be driven primarily by elevated mI, with possible but nonsignificant contributions from lower tNAA. This may reflect the younger age and earlier disease stage of our cohort, in which gliosis is detectable but overt neuronal loss is not yet manifest at the voxel level. Further, since these data were collected at a relatively long TE, optimized for GABA + and GSH editing, some loss of overall signal and precision is expected. Short-TE acquisitions would likely retain more signal and improve quantification of metabolites such as NAA and mI. Finally, the cross-sectional design limits conclusions regarding progression; longitudinal studies are needed to establish whether mI reliably tracks disease progression and treatment response.

## CRediT authorship contribution statement

**William Gaetz:** Writing – review & editing, Writing – original draft, Visualization, Methodology, Investigation, Funding acquisition, Formal analysis, Data curation, Conceptualization. **Muhammad G. Saleh:** Writing – review & editing, Writing – original draft, Validation, Software, Methodology, Formal analysis, Data curation. **Charlotte Birnbaum:** Writing – original draft, Project administration. **Luke Bloy:** Data curation, Formal analysis, Software, Visualization, Writing – original draft. **Timothy P.L. Roberts:** Writing – review & editing, Writing – original draft, Supervision, Methodology. **David R. Lynch:** Writing – review & editing, Writing – original draft, Supervision, Resources, Methodology, Funding acquisition.

## Declaration of competing interest

The authors declare that they have no known competing financial interests or personal relationships that could have appeared to influence the work reported in this paper.

## Data Availability

Data will be made available on request.
